# Advances in Application of Cellulose—MOF Composites in Aquatic Environmental Treatment: Remediation and Regeneration

**DOI:** 10.3390/ijms24097744

**Published:** 2023-04-24

**Authors:** Yehan Tao, Jian Du, Yi Cheng, Jie Lu, Douyong Min, Haisong Wang

**Affiliations:** 1 Guangxi Key Laboratory of Clean Pulp & Papermaking and Pollution Control, College of Light Industry and Food Engineering, Guangxi University, Nanning 530004, China; taoyh1818@dlpu.edu.cn; 2 Liaoning Key Laboratory of Lignocellulose Chemistry and BioMaterials, Liaoning Collaborative Innovation Center for Lignocellulosic Biorefinery, Department of Light Industry and Chemical Engineering, Dalian Polytechnic University, Dalian 116034, China; dujian01@dlpu.edu.cn (J.D.); chengyi18@dlpu.edu.cn (Y.C.); lujie@dlpu.edu.cn (J.L.)

**Keywords:** MOFs, cellulose, composite, water treatment, pollution control

## Abstract

Metal organic frameworks (MOFs) have gained remarkable interest in water treatment due to their fascinating characteristics, such as tunable functionality, large specific surface area, customizable pore size and porosity, and good chemical and thermal stability. However, MOF particles tend to easily agglomerate in nanoscale, thus decreasing their activity and processing convenience. It is necessary to shape MOF nanocrystals into maneuverable structures. The in situ growth or ex situ incorporation of MOFs into inexpensive and abundant cellulose-family materials can be effective strategies for the stabilization of these MOF species, and therefore can make available a range of enhanced properties that expand the industrial application possibilities of cellulose and MOFs. This paper provides a review of studies on recent advances in the application of multi-dimensional MOF–cellulose composites (e.g., aerogels, membranes, and bulk materials) in wastewater remediation (e.g., metals, dyes, drugs, antibiotics, pesticides, and oils) and water regeneration by adsorption, photo- or chemocatalysis, and membrane separation strategies. The advantages brought about by combining MOFs and cellulose are described, and the performance of MOF–cellulose is described and compared to its counterparts. The mechanisms of relative MOF–cellulose materials in processing aquatic pollutants are included. Existing challenges and perspectives for future research are proposed.

## 1. Introduction

The world is experiencing drastic industrialization and urbanization, along with which hazardous pollutants, e.g., heavy or radioactive metal ions, organic dyes, waste oils, organic chemicals, etc., are largely discharged into water. These contaminants result in severe aquatic environmental pollution and seriously threaten the health of humans and aquatic organisms. Thus, concerns about water quality standard and water scarcity have risen greatly, leading to the creation of several materials aimed at achieving acceptable standards of aqueous pollutants by various water remediation technologies involving adsorption, catalysis, membrane separation, and so forth [1].

**Figure 1:**
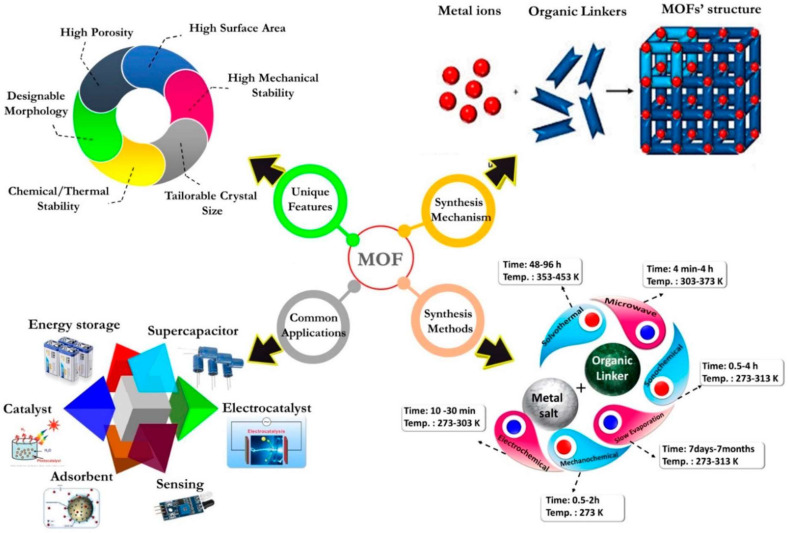
Schematic illustration of the synthesis mechanisms and methods, unique features, and common applications of MOFs (reprinted with permission from Ref. [4], © 2020 MDPI).

Metal organic frameworks (MOFs) are an attractive new class of water remediation material [2]. MOFs are assembled from metal cations or clusters bridged by organic ligands, displaying high crystallinity with reticular topology (Figure 1). The various combinations of metal nodes and organic moieties provide a vast amount of structural and functional diversity within MOFs. The versatile functionalities, together with other fascinating characteristics, including pre-designable pore-size, high surface area (up to 10,000 m^2^/g), high porosity, and homogeneously dispersed and abundant active sites, make MOFs very intriguing candidates for preparing adsorbent, catalyst separation films that can be targeted in the water treatment field [3]. However, intrinsic MOFs exist in powder form, characterized by easy spillage and limited mechanical durability, especially when placed in strong acidic environments; therefore, shaping MOF nanocrystals into various useful configurations remains a longstanding challenge.

**Figure 2:**
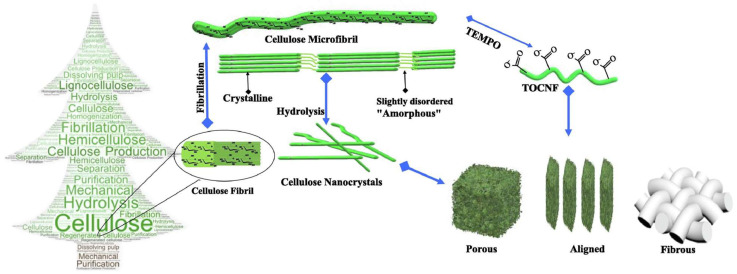
The isolation of cellulose into cellulose nanofibers, cellulose nanocrystals, and TEMPO—oidized cellulose nanofibers (reprinted with permission from Ref. [11], © 2022 Elsevier Inc.).

Cellulose and its derivatives have long been recognized as excellent supports of composites, and, importantly, as a class of effective water treatment materials [5,6,7]. Cellulose is the major constitution of lignocellulose, and can be found in all woody and non-woody plants, as well as other resources, representing the most plentiful and renewable natural polymer on Earth. The utilization of cellulose in the water treatment field is of great importance under the circumstances of rapidly-depleted fossil resources and shortcomings in the world’s developing theme of “Carbon Neutral”. Among the cellulose family, nanocellulose has attracted the most attention (Figure 2) [8]. Nanoscale cellulose fibrils (CNFs) can be readily extracted from a cellulosic source, displaying semi-crystallinity and a high aspect ratio, typically 3–50 nm wide and 1–3 μm long. Hydrolyzing the amorphous parts of the elementary cellulose fibrils can generate cellulose nano whiskers (CNWs), nanorods, or nanocrystals (CNCs), which are highly crystalline (>85%) and possess a low aspect ratio, typically 3–20 nm wide and <500 nm long. The surface of nanocellulose has abundant primary and secondary hydroxyl groups, which offer manifold possibilities for functionalization, such as oxidation, esterification, halogenation, etherification, amination, grafting, and so forth, which is of great interest in preparing various water treatment materials that can satisfy different water remediation requirements [9,10].

Based on the above analysis of MOFs and cellulose materials, it is particularly meaningful to stabilize MOFs with cellulose to develop their unique functional merits and avert their disadvantages in material processing and application. MOFs would improve the flexibility and activity of the composite materials [12]; in turn, the cellulose would help increase porosity, active site accessibility, mechanical stability, and recovery of powdered MOFs [13]. Such MOF–cellulose composites could renovate the industrial application fields of nanocellulose and MOFs. Several reviews have been published targeting the advances in MOF–cellulose composites [11,14,15]. This review emphasizes recent advances (mainly within 5 years) in their applications in the aquatic environmental pollution control field. A summary of the preparation strategies of MOF–cellulose composites is also provided. In the last sections, we summarize the existing challenges in this field and propose some perspectives for future research work.

## 2. Preparation of MOF–Cellulose Composite

**Figure 3:**
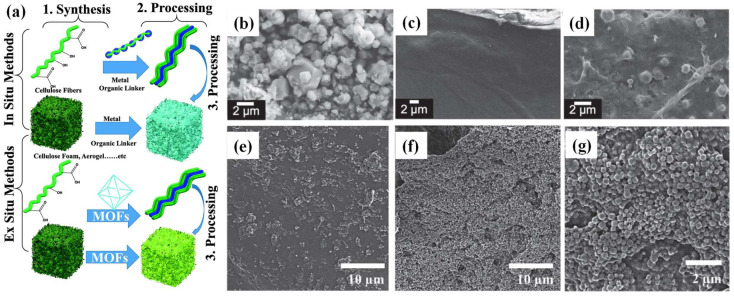
(**a**) Synthesis of MOF–cellulose composites using in situ and ex situ approaches (reprinted with permission from Ref. [11], © 2022 Elsevier Inc.); SEM images of (**b**) intrinsic MOF crystals, (**c**) TOCNF film, (**d**) MOF–TOCNF composite film (adjusted with permission from Ref. [16], © 2015 WILEY-VCH Verlag GmbH & Co. KGaA, Weinheim); SEM images of (**e**) 20 wt% and (**f**,**g**) 50 wt% of UiO-66-containing cellulose aerogels (adjusted with permission from Ref. [18], © 2016 WILEY-VCH Verlag GmbH & Co. KGaA, Weinheim).

For the preparation of MOF–cellulose composites, the MOFs are normally dispersed in the supported phase, while the cellulose, or cellulose derivatives, generally acts as the substrate. The fibrous matrix of cellulose facilitates the in situ growth or ex situ grafting of MOFs to generate various functional composites (Figure 3a).

For the in situ growth of MOFs onto or into cellulosic fabrics, the metal precursor is first anchored to the cellulose surface. Modification of the fiber surface by introducing organic groups promotes the chelation of metal precursors onto the fiber surface. For example, introducing carboxyl groups through carboxymethylation brings about an anionic surface that facilitates the in situ synthesis of MOF crystals. After the fixation of metal centers, the organic moieties and solvents are added to generate MOFs. High-MOF loading can be achieved by creating numerous crystallization nucleation sites. The size of the MOF crystals is controllable due to the steric limitation of the cellulose-based support matrix. The distribution of MOFs can be adjusted by customizing the homogeneity of the anchored metal centers. The attachment of MOFs on cellulose prepared in this way is tight. As far as we know, in 2016, Kitaoka et al. first reported the stabilization of MOFs by in situ growth methods on 2,2,6,6-tetramethylpiperidine 1-oxyl (TEMPO)-oxidated cellulose nanofibers (TOCNF), achieving a 44% MOF loading. The prepared composites were applied in gas separation (Figure 3b–d) [16]. Additionally, in a recent study, it was mentioned that biological MOFs (bio-MOFs) can be prepared using a native or derivative cellulose chain as an organic moiety by the in situ growth method [17].

The second strategy for preparing MOF—cellulose composites is the integration of MOF crystals onto the surface of cellulose-based matrices. The cellulose, in different forms (such as membrane, aerogel, hydrogel, fabric, or paper), was firstly prepared as a substrate, after which MOFs are integrated onto the cellulose matrices. By carefully designing the preparation strategy, the composite can be prepared without sacrificing the intrinsic characteristics of MOFs (e.g., active sites, porosity, and surface area). As far as we know, Zhu et al. firstly reported an MOF–cellulose composite by mixing MOF particles with crosslinkable CNCs and carboxymethyl cellulose (CMC) (Figure 3e–g) [18]. With the development of MOF–cellulose composites, many reports of preparing MOF–cellulose composites, by either in situ or ex situ strategies, have emerged [11,14,15].

## 3. Strategies of Water Remediation and Regeneration by MOF–Cellulose Composites

MOF–cellulose composites are developing quickly as aquatic environmental pollution control materials. In this section, the use of MOF–cellulose composites in different water treatment strategies is reviewed. It should be mentioned that, in this section, some data were reported by the references with exaggerated precision and the reproducibility of these data should be paid attention.

### 3.1. Adsorption

To immobilize and separate hazardous contaminants from wastewater, the most indisputably popular approach is adsorption, which possesses the advantages of simplicity, comparatively low cost, convenience, and efficiency. Adsorbent properties, such as functional groups, the amount and distribution of active sites, pore size, point of zero charge, pore size, and porosity, determine the uptake capacity and sorption kinetics allowing the selective removal of specific contaminants from waste effluent. The economic impacts, such as removal efficiency, working pH, initial ion concentration, adsorbent dosage, adsorption temperature, and operational cost, should be considered when selecting the most suitable working environment.

**Figure 4:**
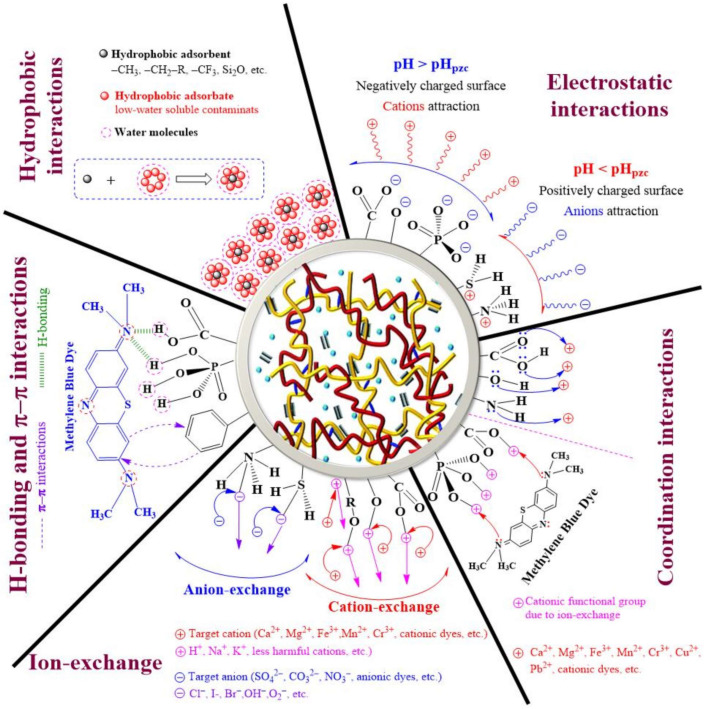
Various interaction mechanisms between adsorbent and adsorbate in wastewater treatment by cellulose–based hydrogels (reprinted with permission from Ref. [5], © 2021 MDPI).

Traditional adsorbents include activated carbon, clay, and ion-exchange resins, though their adsorption capacity and efficiency are not satisfactory. Various MOF–cellulose composites have shown great potential as alternatives for traditional adsorbents in the adsorption of various contaminants. In these composites, the dispersed MOF species offer high porosity and a large surface area, while the cellulose support provides low density, good flexibility, and multi-functionality [19,20]. The porosity of synthetic MOF–cellulose composite materials can reach two or three orders of magnitude higher than some of those above-mentioned traditional materials. Moreover, each repeating unit of the MOF component is tunable and capable of being functionalized to install specific chelating groups suitable for different adsorption requirements. The driving force of adsorption between the composite and the pollutant molecules could be H-bonding, electrostatic forces, π-π interactions, hydrophobia, or acid–base interactions (as shown in Figure 4) [5].

#### 3.1.1. Metal Ion Wastewater

Metal ion pollution generated by industrial production or mineral exploration severely threatens human and environmental health, as these ions are highly mobile in aqueous environments. Therefore, the remediation of heavy metal ion pollution is significant; moreover, the recovery of some valuable metal ions (i.e., lithium for electric batteries), instead of exploiting newly mined ores, is also important. The adsorption strategy is widely reported for the treatment of metal ions in wastewater, and Table 1 summarizes the different MOF–cellulose composites that have been developed as adsorbents [21].

**Figure 5:**
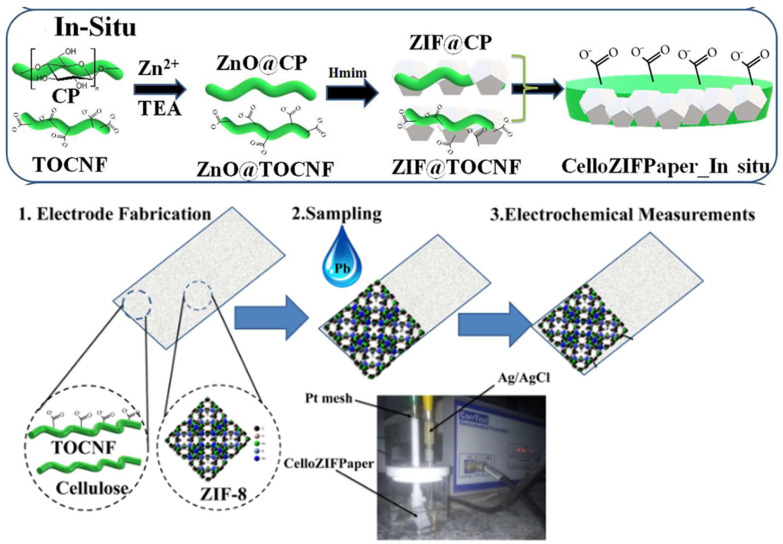
In situ procedure of preparing CelloZIFPaper and schematic representation of electrode fabrication, sampling, and electrochemical measurements of CelloZIFPaper. (TOCNF: TEMPO-oxidized cellulose nanofibrils, ZIF-8: Zeolitic imidazolate framework-8) (adjusted with permission from Ref. [23], © 2022 Elsevier Inc.).

Metal ions include positively and negatively charged cations and anions. A series of Zn-based MOFs, supported on cellulose-based substrate, have been reported for metal ion removal. MCNC@Zn-BTC, which was synthesized by the in situ growth of zinc acetate dihydrate and benzene-1,3,5-tricarboxylic acid through an Et_3_N-catalyzed process on magnetic CNC-wrapped Fe_3_O_4_, was reported to be able to remove Pb(II) cations. Atomic absorption spectrophotometry (AAS) was employed to measure the Pb(II) concentration. The adsorption capacity reached around 559 mg/g within 30 min at 25 °C, and remained greater than 80% after being recycled five times [22]. Another kind of Zn-MOF, ZIF-8, with a hierarchical porous structure, was integrated in situ into a cellulose paper with a diameter of 200 nm using a Rapid Köthen flat sheet former. The prepared CelloZIFPaper composite exhibited adsorption capacities of 66.2–354.0 mg/g toward various heavy metal ions, i.e., Fe^3+^, Cu^2+^, Pb^2+^, Co^2+^, and Cd^2+^ (see Table 1), the concentrations of which were quantitatively analyzed by flame atomic absorption spectroscopy (FAAS). The CelloZIFPaper displayed higher adsorption capacities compared to the reference composite prepared by the ex situ integration of ZIF-8. Interestingly, the CelloZIFPaper was also used in the selective detection of Pb(II) ions by an electric sensing process. The metal ions contained within CelloZIFPaper worked as a stand-alone working electrode, showing a detection limit of 8 μM, with a much lower cost than conventional carbon-based electrodes (as shown in Figure 5) [23]. In another work, ZIF-8 was integrated in situ onto bacteria cellulose, which led to the formation of an aerogel composite with high hierarchical porosity and a large surface area. The aerogel structure endowed the composite with excellent mass transfer productivity. The prepared BC@ZIF-8 composite, with 55 wt% ZIF-8 loading, achieved an 81% adsorption efficiency for Pb^2+^ ions in simulated industrial wastewater after 24 h (the concentration of Pb^2+^ was measure by inductive coupled plasma (ICP) analysis). Its performance was 1.2 times higher than that of the original ZIF-8 nanoparticle counterpart adsorbent. The BC@ZIF-8 composite also displayed an adsorption capacity towards Cd^2+^, with an equilibrium amount of 220 mg/g. The recyclability of the material was not provided [24].

Beyond adsorbing metal cations, a Zn-MOF based adsorbent has also been reported to treat metal anions. Elemental Cr is typically found in effluent streams in the form of anions, including HCrO_4_^−^, CrO_4_^2−^, and Cr_2_O_7_^2−^. With the aim of designing a Cr(VI) removal adsorbent, ZIF-8 particles were entrapped in a porous cellulose aerogel. The reported ZIF-8@CA, with 30 wt% ZIF-8 loading, displayed an adsorption capacity of 27.8 mg/g which fit the pseudo-second-order kinetic model and Langmuir isotherm, better than the intrinsic ZIF-8 particles or cellulose aerogel counterpart adsorbents [26]. In another work, ZIF-8 nanocrystals were generated in situ on kraft pulp, and then mixed with CNF to form a hybrid ZIF-8@CNF@cellulose foam. CNF was employed as a bridge substance, which greatly reinforced the porous structure of the composite foam by hydrogen bonding between the components, leading to a greatly enhanced compression strength of 1.30 MPa, compared to pure cellulose foam. The ZIF-8@CNF@cellulose foam showed a surprisingly fast Cr(VI) adsorption capacity of 35.6 mg/g within 15 s. It should be noted that the adsorption capacity was calculated by comparing the weight of the adsorbent before and after adsorption [27].

A Zr-MOF–cellulose composite was also shown to display an adsorption capacity for metal ions. Amino-functionalized UiO-66 was grafted in situ onto cellulose aerogels at room temperature. The amino functionalization may have favored the adsorption of cations and enhanced the compressive stress of the composite. The equilibrium adsorption capacities of the UiO-66-NH_2_@cellulose aerogel for Pb^2+^ (~89 mg/g) and Cu^2+^ (~39 mg/g) were slightly superior to those of the UiO-66@cellulose aerogel without any modification (81.30 mg/g and 31.23 mg/g, respectively). The reported composite was easily separated without any secondary pollution [25]. In another work, UiO-66 nanoparticles were grafted onto NHNH_2_–CMC and CHO–CNC hybrid modified cellulose aerogels to prepare a UiO-66–CNC–CMC composite, which displayed an 18.2 mg/g adsorption capacity (calculated based on the MOF portion of the aerogel) for Cr(VI) by quantification analysis of the metal concentration using a UV-visible spectrophotometer [18].

**Figure 6:**
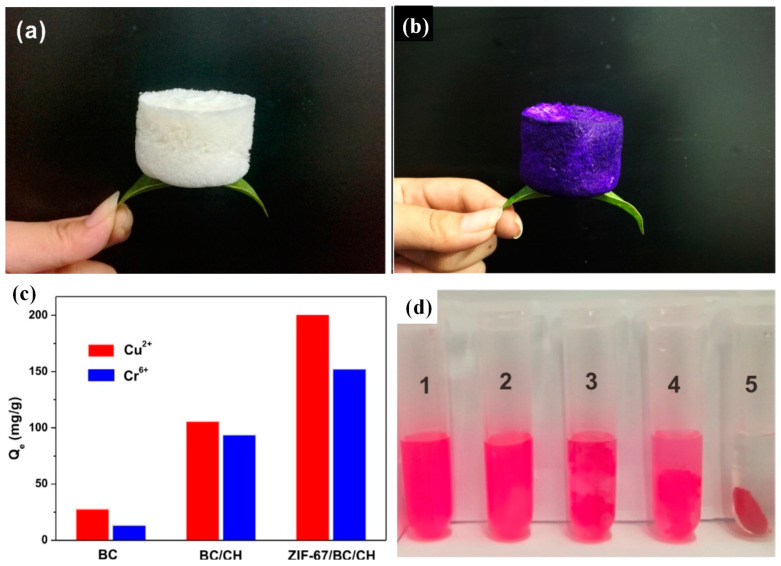
Digital photos of (**a**) pure bacterial cellulose aerogel; (**b**) ZIF-67–bacterial cellulose–chitosan composite aerogel; (**c**) adsorption capacity of bacterial cellulose, bacterial cellulose–chitosan, and ZIF-67–bacterial cellulose–chitosan aerogels for Cu(II) and Cr(VI); (**d**) adsorption of X-3B dye over different samples (sample 5 contains the ZIF-67–bacterial cellulose–chitosan aerogel) (adjusted with permission from Ref. [28], © 2020 Elsevier Inc.).

The above-mentioned Zn–MOFs, or Zr-MOF–based composites, were only reported to treat one type of metal ion (either cations or anions); the development of amphoteric adsorbents is a practical and meaningful research direction for the treatment of industrial wastewater which contains complicated metal ions. Based on this consideration, Co-MOFs (ZIF-67) were in situ grown on a physical mixture of bacteria cellulose and chitosan to prepare a novel composite aerogel (as shown in Figure 6) for zwitterionic metal ion removal. This adsorbent inherited high porosity from the MOF component, reaching a high surface area of 268.7 m^2^/g, much higher than that of the cellulose–chitosan aerogel (8.4 m^2^/g). On the other hand, bacteria cellulose nanofibers provided good mechanical properties and water stability to the composite. After 24 h adsorption, the adsorption capacities for Cu(II) cations and Cr(VI) anions reached 200.6 mg/g and 152.1 mg/g, respectively [28].

#### 3.1.2. Dye Wastewater

Dyes are widely used in daily life and production, with an approximate annual load of 100 tons of discharge into aquatic environments. Dye wastewater, with high mobility and toxicity, has detrimental effects on plants, animals, and even humans. Particularly, some dyes possess complicated structures that make them resistant to degradation and can therefore permanently damage aqueous media.

**Figure 7:**
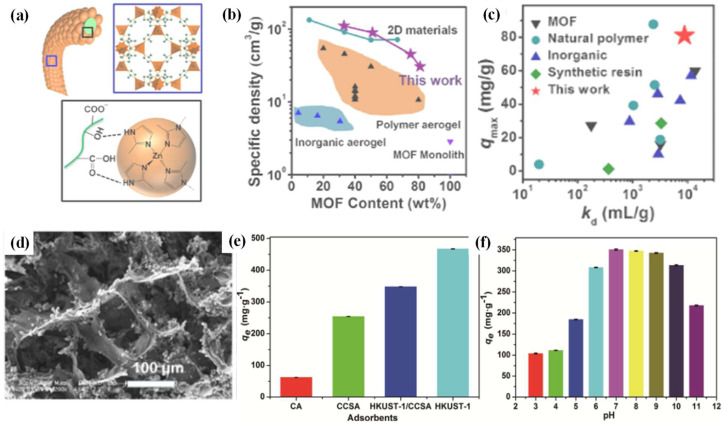
(**a**) Schematic illustration of hydrogen binding between CNFs and ZIF-8 crystals; (**b**) specific density and ZIF-8 contents of MOF aerogels in comparison to other porous materials with MOF loading; (**c**) maximum uptake amount and k_d_ value in comparison to other porous absorbents (adjusted with permission from Ref. [29], © 2018 American Chemical Society); (**d**) SEM image of HKUST-1-CCSA composite aerogel; effects of (**e**) different adsorbents and (**f**) initial pH on the methylene blue adsorption capacity (adjusted with permission from Ref. [31], © 2021 Elsevier Inc.).

Various MOFs with different metal nodes (Zn, Fe, Cu, Co, Zr, etc.) have demonstrated adsorption properties for dye wastewater. For example, ZIF-8 crystals, with high hierarchical porosity of up to 99%, were synthesized onto TEMPO-oxidized CNFs as a template, under the help of which smaller MOF crystals (with a size of 18–65 nm in diameter), with less aggregation and a higher specific density trend, can be obtained (as shown in Figure 7a,b). When applied in Rhodamine B adsorption, the prepared ZIF-8–CNF aerogels, with 33 wt% ZIF-8 loading (~18 nm in diameter), achieved an equilibrium adsorption capacity of 83.3 mg/g and a rapid adsorption rate of 0.036 mg/h, with slightly decreased recyclability. The concentrations of dye were measured by UV-vis spectroscopy. The adsorption properties and distribution coefficient of the ZIF-8–CNF aerogel were superior to many reported natural polymers, as well as some inorganic materials and synthetic resins (Figure 7c) [29]. Another previous study reported a ZIF-8-based adsorbent (ZIF-8@CNF@cellulose foam) which demonstrated a lower adsorption capacity, 24.6 mg/g, for Rhodamine B [27].

Functionalized Fe-MOFs and cellulose-based composite aerogels (NH_2_-MIL–88(b)@CA) were prepared by the in situ growing strategy and applied to the removal of Congo red dye, with an adsorption capacity of 280.3 mg/g and good recycling capability for five cycles. The method of adsorption of Congo red dye by the adsorbent was chemical adsorption, as calculated by the pseudo-second order dynamic model [30].

Cu-based MOFs also displayed an adsorption capacity for dye wastewater. An HKUST-1–cellulose–chitosan composite aerogel (HKUST-1/CCSA) was prepared. The cellulose–chitosan aerogel (CCSA) support was prepared by freeze-drying a hydrogel obtained by covalent cross-linking and the freeze–thaw method. Then, Cu(NO_3_)_2_ and trimesic acid were added to the support to grow the Cu-MOFs, leading to the formation of the final aerogel (Figure 7d). A high chitosan content is beneficial for enhancing the growth of HKUST-1 and surface area, which reached as high as 978.57 m^2^/g. The composite aerogel displayed an adsorption capacity 347.7 mg/g for methylene blue, far superior to its counterpart adsorbents of chitosan and cellulose–chitosan, though a bit lower than the HKUST-1 adsorbent (466.9 mg/g). The initial pH value had a significant effect on the adsorption capacity, with a neutral pH environment being more suitable for dye adsorption (Figure 7e,f) [31].

The above-mentioned amphoteric Co-MOF–bacterial cellulose–chitosan composite aerogel, with 46% ZIF-67 loading (Section 3.1.1), showed an almost 100% favorable remediation rate toward active red X-3B through one instance of adsorption (sample 5 in Figure 6d), while the counterpart cellulose aerogel showed almost no adsorption for the dye (sample 2 in Figure 6d) [28].

As stated above, the development of an amphoteric adsorbent that can treat wastewater with different states of charge is a promising research direction. An amphoteric adsorbent (UiO-66–nanocellulose aerogel) was prepared with UiO-66 self-crosslinked to nanocellulose by hydrogen bonding between the Zr-OH groups of the MOF particles and the hydroxyl groups of the nanocellulose chains. The composite aerogel, with up to 50 wt% Zr-MOF loading, demonstrated good adsorption capacities towards both cationic methylene blue (51.8 mg/g) and anionic methyl orange (71.7 mg/g) via electrostatic, π-π interactions, and hydrogen bonding. [32] Particularly, a zwitterionic adsorbent with binary MOF contents was investigated for amphoteric pollutant adsorption. Anionic HKUST-1 MOFs, cationic ZIF-67 MOFs, and CNF hydrogels were homogeneously mixed in liquid nitrogen, followed by freeze-drying to form a zwitterionic adsorbent. The adsorbent displayed a flexible aero-bead form with a high hierarchical porosity of 98.96% and very low density of 0.015 g/cm^3^. Its adsorption capacity for anionic methyl orange was 49.2 mg/g; owing to its bead-shape appearance, this adsorbent can be easily recovered by filtration for recycling [33].

#### 3.1.3. Pharmaceutical Wastewater

**Figure 8:**
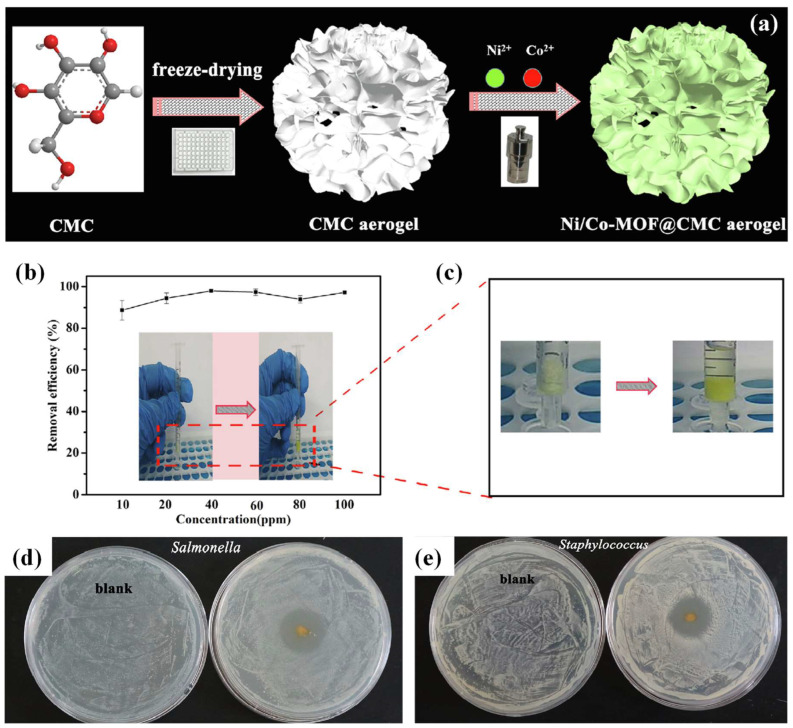
(**a**) Schematic representation of the preparation of Ni/Co-MOF@CMC aerogel; (**b**) effect of tetracycline concentration on removal efficiency using the Ni/Co-MOF@CMC aerogel and the photograph of the filter; (**c**) color changes of the Ni/Co-MOF@CMC aerogel before and after adsorption of tetracycline; antibacterial effect of (**d**) Salmonella and (**e**) Staphylococcus aureus by tetracycline-adsorbed Ni/Co-MOF@CMC aerogel (adjusted with permission from Ref. [34], © 2019 Elsevier Inc.).

Emerging pharmaceutical pollution with toxic, carcinogenic, and mutagenic effects can aggravate mortality in aquatic organisms and even humans. For example, diclofenac (a commonly used anti-inflammatory drug), the fatal effects of which have been proven on wild animals of the Indian subcontinent [33]. A few binary MOF species containing composites have been reported for the removal of pharmaceuticals. For example, the above-mentioned zwitterionic Cu-Zn-MOFs@CNF aero-beads, which combined binary HKUST-1 MOF and ZIF-67 MOF components (in the last part of the Section 3.1.2), showed a high adsorbing capacity of 121.2 mg/g towards the anionic pharmaceutical diclofenac [33]. Binary Ni/Co-MOFs were introduced onto the CMC aerogel substrate for tetracycline hydrochloride removal (Figure 8a–c). The CMC aerogel template offered a highly porous 3D structure that allowed the continuous shipping of tetracycline hydrochloride solution, and facilitated the interaction between tetracycline hydrochloride and Ni/Co-MOF via prolonged residence time. Within 5 min, tetracycline hydrochloride was rapidly remediated by the composite aerogel, with an excellent adsorption capacity of up to around 625 mg/g and a removal rate of around 80%. The concentrations of the tetracycline were monitored by a UV-2500 spectrophotometer. Interestingly, the tetracycline hydrochloride-adsorbed Ni/Co-MOF@CMC aerogel could act as an efficient bacteriostatic agent against Salmonella and Staphylococcus aureus (Figure 8d,e) [34].

**Figure 9:**
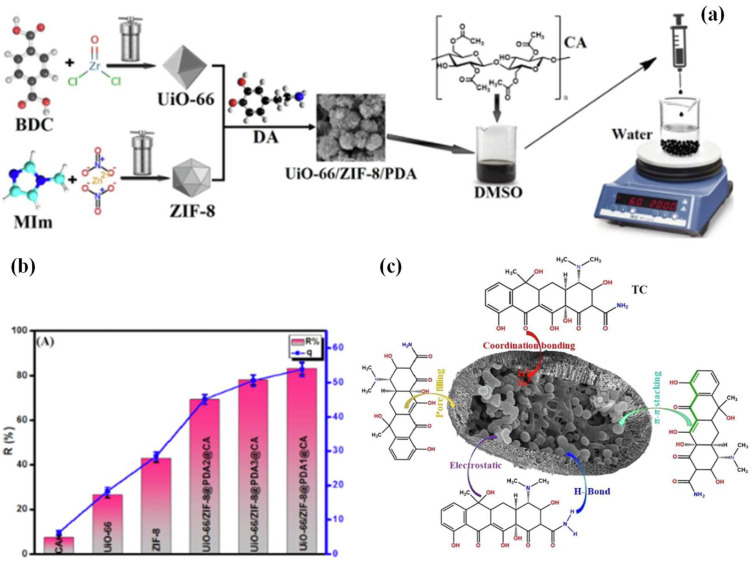
(**a**) Schematic representation of the synthesis of UiO-66/ZIF-8/PDA/CA composite beads (The atoms of different color balls in BDC: gray: C, red: O, white: H, blue: N; in MIm: light blue: C, dark blue: N, white: H; in DA: dark: C, red: O, white: H, blue: N); (**b**) comparison between different adsorbents; (c) plausible adsorption mechanism of TC by UiO-66/ZIF8PDA@CA (adjusted with permission from Ref. [35], © 2022 Elsevier Inc.).

In addition, zwitterionic, cationic, and anionic Zn-MOFs supported on polydopamine and cellulose acetate composites (UiO-66/ZIF-8/PDA@CA) were utilized to decontaminate tetracycline pollution. The composite was fabricated in the form of beads, with a large specific surface area of 118.7 m^2^/g (as shown in Figure 9a). When applied to the adsorption of tetracycline, the adsorbent displayed a good adsorption capacity of around 290.7 mg/g within 60 min at 25 °C. The bead shape endowed the adsorbent with easy separation, while the adsorption efficiency was retained for 67% after eight cycles of adsorption–desorption. According to the mechanism analysis, the adsorption process of tetracycline relied on pore filling, π-π interactions, H-bonding, and coordination bonding (as shown in Figure 9b,c) [35].

In addition to binary MOFs, single MOF species, e.g., ZIF-67, were also grown on polyaniline-modified regenerated cellulose aerogels to remove tetracycline from aquatic systems and decontaminate tetracycline pollution. Polyaniline-coated regenerated cellulose aerogel played the role of metal-chelated layers and effectively promoted the in situ growth of ZIF-67, reaching a ZIF-67 loading mass ratio of 25.39%. The obtained composite adsorbent demonstrated a high tetracycline adsorption capacity of 409.5 mg/g, and remained over 94% efficiency after six reuse cycles [36].

The capture of radio iodine from nuclear-related activities is also of great significance. A (Co-Fe)^II^(pz)[Ni^II^(CN)_4_] MOF–cellulose hybrid aerogel (CoFe@CA-D) was synthesized by the doping method and applied in the adsorption of iodine from an aqueous environment. By utilizing the doping strategy, the loading of (Co-Fe)^II^(pz)[Ni^II^(CN)_4_] MOF nanoparticles reached 45.8%, with a high porosity of 87.8%. The equilibrium adsorption capacity for tetracycline achieved around 458 mg/g, as tested by UV-vis analysis, with good reusability for five cycles [37].

### 3.2. Catalysis

As discussed in Section 3.1, heavy metal ions, organic dyes, and pharmaceutical wastewater can have a devastating impact on the aqueous environment, as well as animal and human health. Therefore, the treatment of these pollutants should be more complete than simple adsorption. For example, advanced catalysis, with simplicity, high efficiency, and good reproducibility, has been considered as a promising technology for converting or degrading organic pollutants into compounds with lower or no toxicity. Among different advanced catalysis process, photocatalysis and chemocatalysis have been reported to be applied in MOF–cellulose composites for aquatic environmental pollution control.

#### 3.2.1. Photocatalysis

Photocatalysis reactions rely on the excitation of a photocatalyst, which can absorb photo radiation and release photo-induced electrons to motivate photocatalysis reactions [38]. Typical photocatalysts involve metal oxide semiconductors and a nonmetallic organic photocatalyst. TiO_2_ is the most widely used photocatalyst because of its superior UV radiation absorption capacity, good thermal and chemical stability, and economic efficiency. Ti-based MOFs have also been reported as photocatalysts. Amino-functionalized Ti-MIL-NH_2_ was inserted within a cellulose acetate film to prepare a porous composite film (CA@Ti-MIL-NH_2_); this film was evaluated in the degradation of paracetamol, which is one of the most commonly used intermediates in pharmaceutical industries. The degradation kinetic parameter (k_1_ = 760.0 m^−1^) tested much higher than the adsorption kinetic value (k_1_ = 160.0 m^−1^), which was ascribed to the high photocatalytic activity of the Ti-based component. After photo degradation, the paracetamol was completely converted to carbon dioxide and water, according to the results of an organic carbon tracking experiment. The concentrations of paracetamol were analyzed by a UV-vis spectrometer at 243 nm. The removal efficiency of the porous CA@Ti-MIL-NH_2_ film achieved 519.1 mg/g, much greater than that of the intrinsic CA film (82.7 mg/g), due to the photodegradation performance of the CA@Ti-MIL-NH_2_ film [39].

**Figure 10:**
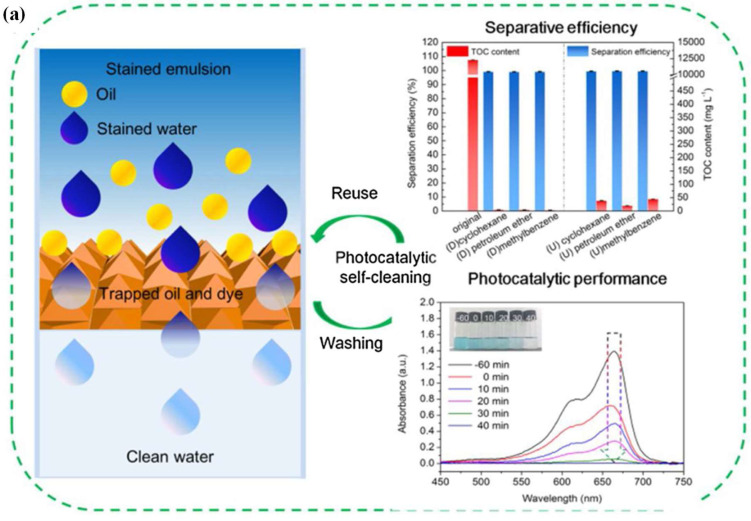

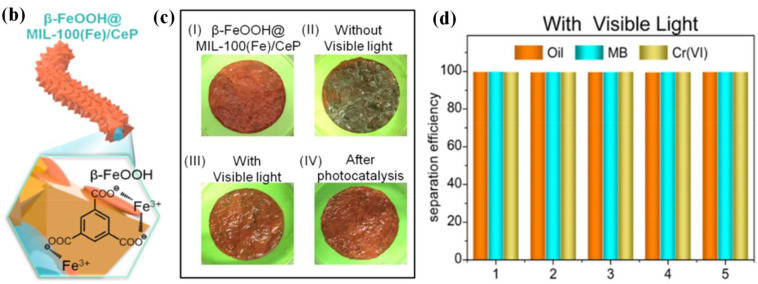
Fe-MOF—based photocatalysts: (**a**) performance of oil separation and photocatalytic degradation of dye, with mechanisms (adjusted with permission from Ref. [40], © 2020 Elsevier Inc.); (**b**) schematic diagram of core–sheath structured β-FeOOH@MIL-100(Fe) −CeP; (**c**) photography of the composite in different stages; (**d**) removal rate of oil/MB/Cr(VI) in cyclic experiment (adjusted with permission from Ref. [41], © 2021 Elsevier Inc.).

In addition to Ti-MOFs, a few Fe-MOF-based composites have been reported as photocatalysts for wastewater treatment. For example, Ni et al. prepared a MIL-100(Fe)-based composite and applied it in the photocatalytic remediation of wastewater containing complex pollutants (dye and oil) (as shown in Figure 10a). For the synthesis, the MIL-100(Fe) was grown in situ on carboxymethylated cotton fabric, followed by immobilizing Ag@AgCl nanoparticles to prepare the Ag@AgCl@MIL-100(Fe)-CCF composite membrane. The cotton fabric support underwent a carboxymethylation process to improve the hydrophilicity of the fabric, which facilitated the growth of Fe-MOFs and further enhanced the adsorption performance. The addition of Ag@AgCl nanoparticles was intended to increase the photocatalytic activity of the composite membrane. The prepared membrane simultaneously decontaminated the wastewater of oil and dyes, with removal efficiencies of 99.64% and 97.3%, respectively, and had good recyclability. Additionally, the composite membrane degraded various organic dyes (methylene blue and rhodamine B) in the presence of O_2_ within a short reaction time under visible light irradiation [40]. The group further developed a MIL-100(Fe)-MOF-based membrane with a core-sheath structure for photocatalytic wastewater treatment. Deacetylated cellulose acetate-PVP was selected as the support for in situ grown MIL-100(Fe), after which β-FeOOH was immobilized onto the support by an impregnation method, displaying a homogeneous β-FeOOH nanorod dispersion with a large surface area of 1105 m^2^/g. Following a photocatalysis–Fenton synergy mechanism from (β-FeOOH@MIL-100(Fe), the as-prepared membrane decontaminated wastewater containing insoluble oil, soluble dye, and heavy metals, with removal rates over 99% and good reusability for each compound under visible light (as shown in Figure 10b–d) [41]. Liu et al. prepared MIL-101 Fe-MOF-based cellulosic foam for photocatalytic heavy metal ion removal. The Fe-MOF was amine-functionalized with the aim of enhancing adsorption capacity. The Cr(VI) pollutant was completely removed by the composite under visible light at a pH of 2, and the efficiency remained over 80% after 10 cycles [42].

#### 3.2.2. Chemocatalysis

**Figure 11:**
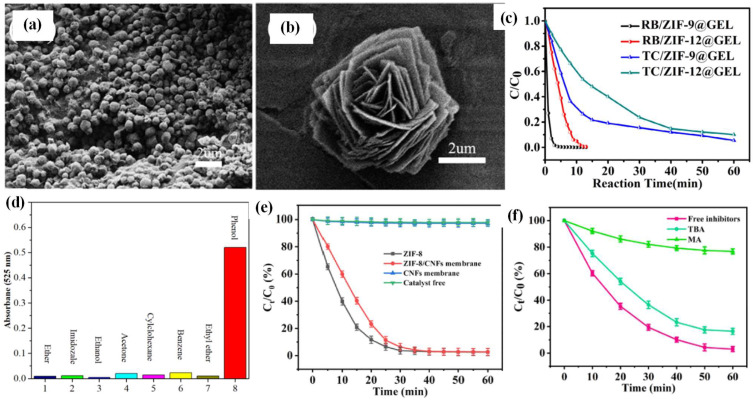
SEM images of (**a**) ZIF-9@GEL, (**b**) ZIF-12@GEL, and (**c**) their degradation performance (adjusted with permission from Ref. [45], © 2018 Elsevier Inc.); (**d**) absorbance value of Co_3_O_4_/CDM at 525 nm of the solutions containing 70 μM phenol or various species (adjusted with permission from Ref. [46], ©2021 Elsevier Inc.); (**e**) decolorization results of methyl blue with PMS activated by different materials with pH 7 at 25 °C; (**f**) effect of inhibitor on the degradation of methylene blue with pH 4 at 25 °C (adjusted with permission from Ref. [47], © 2022 Elsevier Inc.).

A series of MOF–cellulose composites were reported to adsorb aquatic pollutants and further degrade them into non-toxic compounds by chemocatalysis oxidation or reduction methods [43]. A typical process is the advanced oxidation processes (AOPs). Various Co-MOF-based composites have been reported to catalyze AOPs. For example, a ZIF-67–cellulose hybrid membrane was fabricated by the in situ growth method for degrading organic contaminants in sulfate radical-based advanced oxidation processes (SR-AOPs). The membrane, containing Co catalytic centers, was able to catalytically activate peroxymonosulfate (PMS) to generate SO_4_^−^ and OH, resulting in the rapid degradation of methylene blue dye and Rhodamine B dye into low-molecular weight substances, further degrading into CO_2_ and H_2_O within 1 min. The hybrid membrane worked effectively in a wide pH range of 4–9, with good stability and reusability [44]. In another work, ZIF-9 or ZIF-12 were separately loaded on cellulose aerogel (as shown in Figure 11a,b) to activate PMS for the degradation of various pollutants, e.g., Rhodamine B, tetracycline hydrochloride, and p-nitrophenol (PNP). The composite displayed an almost complete removal rate for dye within 10 min, and a 90% removal rate for antibiotics within 1 h (see Figure 11c). Even applied in the removal of stubborn PNP, the hybrid aerogel displayed an over 90% remediation rate within 1 h, and retained its activity in a wide pH range with good reusability. The concentration of PNP was analyzed by a UV-vis spectrometer [45]. In another work, Co-MOF-74 was grown in situ on a regenerated cellulose membrane. By applying hydrothermal operation, the loaded Co-MOF-74 was recrystallized, after which annealing of the composite led to the formation of final product, a Co-MOF-derived Co_3_O_4_-based membrane catalyst (Co_3_O_4_/CDM), which was used for the detection and elimination of phenol. The prepared composite accomplished fast colorimetric detection of phenol in the presence of H_2_O_2_ and 4-aminoantipyrine, with a low detection limit and high selectivity over a serious of other compounds (Figure 11d). Meanwhile, within 20 min, the Co_3_O_4_/CDM catalytically eliminated over 90% of the phenol with the help of PMS [46].

In addition to Co-MOFs, Zn-MOFs (ZIF-8) were immobilized onto CNFs to prepare a catalytic composite membrane for the degradation of dyes. The Zn centers activated PMS to generate free SO_4_^−^ and OH radicals. ZIF-8–CNF composite membrane degraded over 95% of the methylene blue and/or Rhodamine B in the presence of PMS within 60 min (Figure 10e). Cycle test results showed that the composite membrane has outstanding recycling performance, retaining excellent degradation performance even after five cycles. Mechanism analysis, performed by adding inhibitors, demonstrated that SO_4_^−^ played a more dominant role than OH in the removal of methylene blue and Rhodamine B (as shown in Figure 11f) [47].

Another kind of oxidation process is based on the Fenton reaction. A composite of cellulose micro-fibrils supporting Cu-MOF (HKUST-1) crystals and Fe_3_O_4_ nanoparticles (HKUST-1/Fe_3_O_4_/CMF) were prepared for the degradation of dye in a catalytic Fenton system. The synthesized composite displayed good adsorption performance due to the integration of porous Cu-MOFs, and displayed catalytic activity in the Fenton system due to the anchor of Fe_3_O_4_ nanoparticles on the CMF support. An adsorption capacity of 68% with a degradation rate of around 98% for methylene blue was achieved, higher than that of the HKUST-1/Fe_3_O_4_ composite without CMF support (41%) [48].

**Figure 12:**
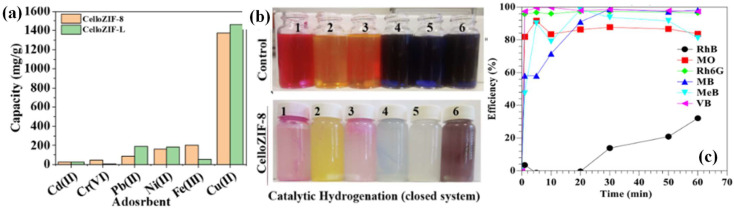
(**a**) Metal adsorption onto CelloZIF materials; (**b**) original dye solutions and dye solution after catalysis in a closed system using CelloZIF-8 (1: RhB, 2: MO, 3: Rh6G, 4: MB, 5: MeB, 6: VB); (**c**) kinetics of catalytic hydrogenation using CelloZIF-8 (reprinted with permission from Ref. [49], © 2022 Elsevier Inc.).

In addition to oxidation processes, catalytic reduction reactions, e.g., hydrogenation, can also help the complete elimination of organic pollutants. With the aim of degrading dye via hydrogenation, ZIF-8 was grown in situ on a TOCNF template in water at room temperature to prepare a CelloZIF-8 composite. Different synthetic parameters were investigated, including the addition of NaOH, the order in which the chemicals were added, and the molar ratio of the reactant. On one hand, the prepared composite was utilized as an adsorbent for different metals, with the concentrations of the metals monitored by AAS or ICP-OES (as shown in Figure 12a). On the other hand, the prepared CelloZIF-8 composite exhibited full removal efficiency towards methyl blue, with reasonable selectivity and a remarkable degradation efficiency of 100% by NaBH_4_ hydrogenation in a closed system (sample 5 in Figure 12b). In addition to anionic methyl blue, CelloZIF-8 was also able to catalytically hydrogenate a few cationic dyes (as shown in Figure 12b,c). The concentration of dyes were measured by a UV-vis spectrometer, while the hydrogen generated during hydrogenation was measured by the water-displaced method [49].

A Cu-MOF-derived catalyst was also prepared for the catalytic reduction of pollutants in the presence of NaBH_4_ by direct annealing of the HKUST-1/Fe_3_O_4_/CMF composite. This composite acted as a catalyst for the catalytic hydrogenation reduction of methyl blue, methyl orange, and 4-nitrophenol, with nearly 100% degradation efficiency and reusability. This superior catalytic performance was brought about by a high surface area and the chemical stability generated by CMF-derived carbon [50]. Another Cu-MOF-based nanohybrid catalyst was reported for the catalytic reduction of 4-nitrophenol by NaBH_4_. The catalyst was prepared by immobilizing silver nanoparticles onto MOF-199s supported by carboxymethylated cellulose fibers (Ag NPs@MOF-199s/CCFs). The as-prepared nano catalyst showed a high kinetic rate constant and turnover frequency (compared to counterpart catalysts) of 2.48 × 10^−3^/s and 559.2/h, respectively, which can be ascribed to the well-dispersed silver nanoparticles on the MOF-199s and the porous structure. The catalyst displayed a stable reusability after five runs [51].

An MOF–cellulose catalyst was reported to enable the detoxification of pollutants by a catalytic hydrolysis process. A Zr-MOF-loaded functionalized cellulose sponge was applied for rapid hydrolysis of 4-nitrophenyl phosphate, the half-time of which was as short as 9 min. The rapid catalytic activity was attributed to its high surface area, high porosity, good mechanical strength, and good thickness recovery [52].

### 3.3. Membrane Separation

Membrane technology remains one of the most convenient and efficient ways to remove aquatic contaminants in one step, via size exclusion and/or adsorption [53]. The addition of MOFs in composite membranes provides benefits from their intrinsic properties, including customizable modularity and functionality, high specific surface area, and accessible voids [54]. Meanwhile, the introduction of low-cost, abundant, and biodegradable cellulose into water treatment membranes undisputedly increases the economic efficiency and sustainability of this process [55,56]. Significant selective and effective separation of various pollutants can be achieved by the application of MOF–cellulose membranes.

**Figure 13:**
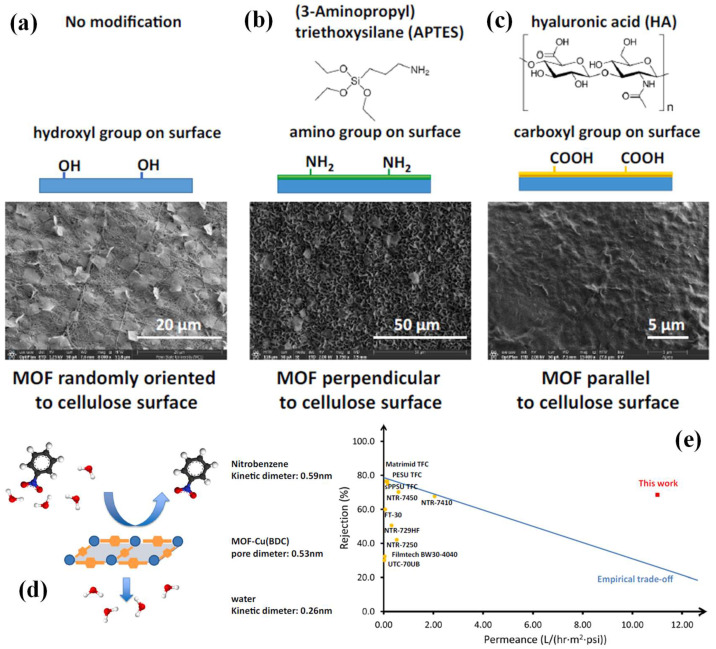
(**a**–**c**) Surface modification agents decide MOF orientation on surface; (**d**) MOF Cu(BDC) pore diameter in between nitrobenzene and water kinetic diameter result in high rejection rate towards nitrobenzene (Explanation of different colors of balls: black: C, blue: N, red: O, white: H); (**e**) schematic of membrane trade-off performance (adjusted with permission from Ref. [57], © 2021 Elsevier Inc.).

Cu(BDC) MOFs were coated on bacterial cellulose to prepare a separation membrane for nitrobenzene. The orientation of the MOFs (random, parallel, or perpendicular, as seen in Figure 13a–c) on the support was controlled by different coatings on the bacteria cellulose, including hydroxyl, amino, and carboxyl group functionalization. The optimal separation performance, with a high water permeance of 10.85 L/(hr·m^2^·psi) and high nitrobenzene rejection of 68.6% (higher than many pure polymer membranes, as seen in Figure 13d,e), was achieved by parallel orientation, obtained by hyaluronic acid modification [57].

A Cu-MOF-based membrane for decontaminating pesticide wastewater was also developed by the in situ growth of Cu-BTC on cellulose acetate. When the loading of Cu-BTC reached 40 wt%, the surface area of the porous cellulose acetate membrane was greatly enhanced, from 78.4 m^2^/g to 965.8 m^2^/g, leading to a simultaneous increase in adsorption capacity, from 207.8 mg/g for the cellulose acetate membrane to 321.9 mg/g for the Cu-BTC@CA membrane. The applied membrane retained around 78% efficiency over five cycles [58]. Another kind of Cu-MOFs, namely HKUST-1, was blended with graphene oxide (GO) and cellulose acetate through the typical phase inversion process to prepare ultrafiltration membranes. The addition of GO greatly increased the hydrophilicity of the membrane, while the HKUST-1 avoided the stacking problems of GO. The composite membrane achieved a water permeate flux of 183.51 L/m^2^/h under a starting pressure of 150 kPa and a protein solution flux of 109.52 L/m^2^/h, better than the intrinsic cellulose acetate membrane and the counterpart cellulose acetate–GO membrane. The composite membrane also possessed good antifouling performance, evaluated by its flux recovery ratio, which reached 88.13% due to the compact water barrier formed on the hydrophilic surface of the composite, which prevented the adsorption of contaminants [59].

Aluminum fumarate MOF-based membranes were developed for separating pollutants. For example, De et al. incorporated aluminum fumarate in cellulose acetate phthalate as a base polymer to prepare a mixed-matrix membrane for selective adsorption of fluoride. The membrane continuously worked for 17 h for a feed fluoride concentration of 10 mg/L, using a continuous cross flow setup with an equilibrium adsorption capacity of 179 mg/g and a fluoride rejection rate of more than 99%. Additionally, the developed membrane was successful in separating alkalinity, hardness, iron, total organic carbon, and total dissolved solids with 50–90% removal efficiencies [60]. In another work, an aluminum fumarate MOF mixed with chitosan was coated on cellulose acetate to prepare a forward osmosis membrane for nutrients recovery. The application of AlFu-MOFs led to a higher hydrophilicity, and resultant higher water flux and lower reverse salt flux of the membrane, compared to the cellulose acetate or cellulose acetate–chitosan membranes. The composite membrane, with 0.06 wt% AlFu-MOF and 1% chitosan coating, displayed a real wastewater flux of 8.75 LMH [61].

ZIF-8 was grown in situ on TOCNF to prepare a dye wastewater purification membrane (ZIF-8–TOCN). The ZIF-8–TOCN membrane, with 20 μm thickness, maintained a superior water flux of 84 Lm^−2^/h/bar for 24 h under a pressure range of 1–3 bar. Remarkably, due to the negatively charged TOCNF networks, cationic dyes (Janus Green B and methylene blue) were highly selectively removed through strong electrostatic interactions, while neutral dyes (vitamin B12 and bromothymol blue) were almost completely rejected by the ZIF-8–TOCN membrane [62]. Additionally, ZIF-8 was reported to be incorporated ex situ onto cellulose acetate to prepare a forward osmosis membrane for seawater desalination. The content of ZIF-8 and the operation temperature values of coagulation bath, mixing, and annealing were adjusted to enhance the structural and adsorption properties of the composite membrane. The reverse salt flux and water flux of the composite membrane reached 2.84 g/m^2^/h and 50.14 L/m^2^/h, respectively [63].

A Zr-MOF–cellulose fiber-based composite membrane was synthesized for the adsorption removal of dyes. The surface of the cellulose fibers was carboxylation functionalized to create more anchoring sites, which facilitated the grafting of nanocrystalline UiO-66-NH_2_ for the formation of a continuous and homogeneous coating on the support. The membrane decontaminated anionic methyl orange dye with 84.5% efficiency and removed Cr(VI) with 78.2% efficiency using an in-line filtration setup [64].

## 4. Future Perspectives

Cellulose is utilized as a support in MOF–cellulose composites. There are several ways to pretreat biomass to produce cellulose, for example, base or acid treatment. It should be noted that, although biomass is low cost, the cost of producing nanocellulose is high. The mild, green, large-scale, and economic synthesis of nanocellulose as a support for MOF–cellulose composites is still challenging. Exploring a new synthetic route that could directly use cellulose-based sources, e.g., pulp slurry or wood, would help to decrease the cost of the process and promote the commercialization of these composites.

Future investigation about the synthesis of MOFs and cellulose with as-yet-uncharted components for the design and synthesis of new MOF–cellulose composites targeting a broader range of effective, stable, and reusable water treatment processes could be promising. Additionally, the current synthetic approaches can be further optimized by either improving the preparation protocols or combining them with novel synthetic strategies, with the aim of preparing composites in which the MOF species are loaded with appropriate content and dispersion.

Moreover, various combinations of water treatment technologies should be considered, and the process must be designed toward a marketable end product when valuable contaminants are involved. Future work should also attend to the application of MOF–cellulose materials, or combination with other components, for practical applications. The stability in different pH conditions and recyclability of MOF–cellulose composites should be carefully evaluated when processed for commercialization.

Last, but not least, advances towards a deeper understanding of the mechanisms of water treatment process by MOF–cellulose composites are expected. For example, pseudo-second-order is frequently invoked in the discussion of adsorption kinetics, although its validity is strongly debated, as a widely used linear equation is reported to result in spurious correlations for typical experimental data of the adsorption kinetic process. [65,66].

## 5. Conclusions

In this review, recent progress in the application of MOF–cellulose composites in the field of aquatic environmental pollution control was presented and discussed. These composites, in forms ranging from hydrogels, aerogels, bulk materials, and membranes, were utilized as adsorbents, catalysts, or separation membranes to conduct water treatment processes. By combining MOFs and cellulose compositions, most of these materials displayed enhanced physicochemical properties (e.g., specific surface area, porosity, numbers of accessible active sites, and controllable functionality), typically leading to improved water treatment performance compared to counterpart single-component materials. The recyclability of the composites varied between different composites. The challenges in the acquisition of raw nanocellulose material, the prospective outlooks on the improvement of current synthetic strategies, the industrialization and commercialization of MOF–cellulose composites, and the expected deeper understanding of the involved mechanisms were discussed.

## Figures and Tables

**Table 1:** A comparison of different MOF–cellulose composites for heavy metal ion adsorption.

Adsorbent Name	Form	Adsorption Temperature ^a^ and Time	Adsorption Capacity for Different Metal Ions (mg/g)	Ref.
MCNC@Zn–BTC	Bulk	25 °C, 0.5 h	Pb(II): 558	[22]
CelluZIFPaper	Paper	RT, 12 h	Pb(II): 261, Cd(II): 143, Cu(II): 143, Co(II): 350, Fe(II): 354	[23]
BC@ZIF–8	Aerogel	RT, 24 h	Pb(II): 380, Cd(II): 220	[24]
UiO–66–NH_2_@CA	Aerogel	RT, 32 h	Pb(II): 89, Cu(II): 39	[25]
ZIF-8@CA	Aerogel	RT, 2 h	Cr(VI): 28	[26]
ZIF-8@CNF@cellulose	Foam	RT, 15 s	Cr(VI): 36	[27]
UiO-66–CNC–CMC	Aerogel	RT, 24 h	Cr(VI): 18 ^b^	[18]
ZIF-67/BC/CH	Aerogel	RT, 24 h	Cu(II): 200, Cr(VI): 152	[28]

^a^ RT: room temperature in this row was determined by the original research paper. ^b^ The adsorption capacity of the MOF portion of the aerogel.

## Data Availability

Not applicable.
